# PBPK-based translation from preclinical species to humans for the full-size IgG therapeutic efalizumab

**DOI:** 10.3389/fphar.2024.1418870

**Published:** 2024-10-01

**Authors:** Maria Franz, Ravi Kumar Jairam, Lars Kuepfer, Nina Hanke

**Affiliations:** ^1^ Translational Medicine and Clinical Pharmacology, Boehringer Ingelheim Pharma GmbH and Co. KG, Ingelheim, Germany; ^2^ Institute for Systems Medicine with Focus on Organ Interaction, University Hospital RWTH Aachen, Aachen, Germany

**Keywords:** efalizumab, raptiva, cross-species translation, physiologically based pharmacokinetic modeling (PBPK), monoclonal antibody (mAb), target mediated drug disposition (TMDD), rabbit, monkey (Min.5-Max. 8)

## Abstract

**Introduction:**

Animal models play a vital role in pharmaceutical research and development by supporting the planning and design of later clinical studies. To improve confidence and reliability of first in human dose estimates it is essential to assess the comparability of animal studies with the human situation. In the context of large molecules, it is particularly important to evaluate the cross-species-translatability of parameters related to neonatal fragment crystallizable receptor (FcRn) binding and target mediated drug disposition (TMDD), as they greatly influence distribution and disposition of proteins in the body of an organism.

**Methods:**

Plasma pharmacokinetic data of the therapeutic protein efalizumab were obtained from literature. Physiologically based pharmacokinetic (PBPK) models were built for three different species (rabbit, non-human primate (NHP), human). Target binding was included in the NHP and human models. The assumption of similar target turnover and target-binding in NHP and human was explored, to gain insights into how these parameters might be translated between species.

**Results:**

Efalizumab PBPK models were successfully developed for three species and concentration-time-profiles could be described appropriately across different intravenously administered doses. The final NHP and human models feature a common set of parameters for target turnover and drug-target-complex internalization, as well as comparable target-binding parameters. Our analyses show that different parameter values for FcRn affinity are crucial to accurately describe the concentration-time profiles.

**Discussion:**

Based on the available data in rabbits, NHP and humans, parameters for FcRn affinity cannot be translated between species, but parameters related to target mediated drug disposition can be translated from NHP to human. The inclusion of additional pharmacokinetic (PK) data including different efalizumab doses would further support and confirm our findings on identifying TMDD and, thus, binding kinetics of efalizumab in NHPs. Furthermore, we suggest that information on target expression and internalization rates could make it possible to develop comprehensive human PBPK models with minimal animal testing. In this project, we compared the pharmacokinetics of a therapeutic protein in rabbit, NHP and human using an open PBPK modeling platform (Open Systems Pharmacology Suite, http://www.open-systems-pharmacology.org). Our findings could support similar translatory studies for first in human dose predictions in the future.

## 1 Introduction

Animal models play a vital role in pharmaceutical research and development, offering invaluable insights into pharmacokinetics, efficacy and safety before clinical first in human studies. However, the predictive translation between preclinical species and human is still a difficult task. Simple approaches like allometric scaling often do not provide adequate predictions (Ward et al., 1999; Mahmood, 2009; Jairam et al., 2018). A more mechanistic tool to support inter-species scaling is physiologically based pharmacokinetic (PBPK) modeling (Kuepfer et al., 2016). It allows a more comprehensive description and, therefore, translation of the absorption and disposition of a drug, taking into account the physico-chemical properties of the drug, as well as the anatomy and physiology of the considered species. This requires prior knowledge or assumptions on drug-specific parameters; the physiological differences between the species on the other hand are provided in PBPK software tools like PK-Sim which include information on whole-body physiology (Willmann et al., 2005; Kuepfer et al., 2016; Thiel et al., 2015).

There have been attempts to build PBPK models which are able to translate between species, especially for small molecules, as summarized by Thiel et al. (Thiel et al., 2015). Large molecules have been studied to a lower extent in this regard. Shah and Betts et al. developed PBPK models for intravenously administered monoclonal antibodies (mAbs) in mouse, rat, monkey and human for similar, but not-identical compounds, probably due to the scarcity of available data (Shah and Betts, 2012). Two studies by Sepp et al. showed the translation of a mAb PBPK model from rodents to primates for the same compound in each case (Sepp et al., 2020). Pasquiers et al. recently published a cross-species PBPK approach for bevacizumab translating from monkey to human (Pasquiers et al., 2023).

Rabbits are a species which is widely used in animal testing, and particularly in development of drugs for ocular complications (e.g., age-related macular degeneration, diabetic retinopathy), the translation from rabbit to non-human primate (NHP) is common. Naware et al. recently published an ocular PBPK model for rabbit, monkey and human following systemic and intravitreal administration (Naware et al., 2023). However, the datasets selected for this study had linear pharmacokinetics (PK), such that target-binding effects were minimal. Thus, the translation of target-binding effects was not investigated yet. Target-binding of mAbs in rabbits can be weaker compared to humans, e.g., for bevacizumab (EMEA, 2005), or even be not apparent.

**FIGURE 1:**
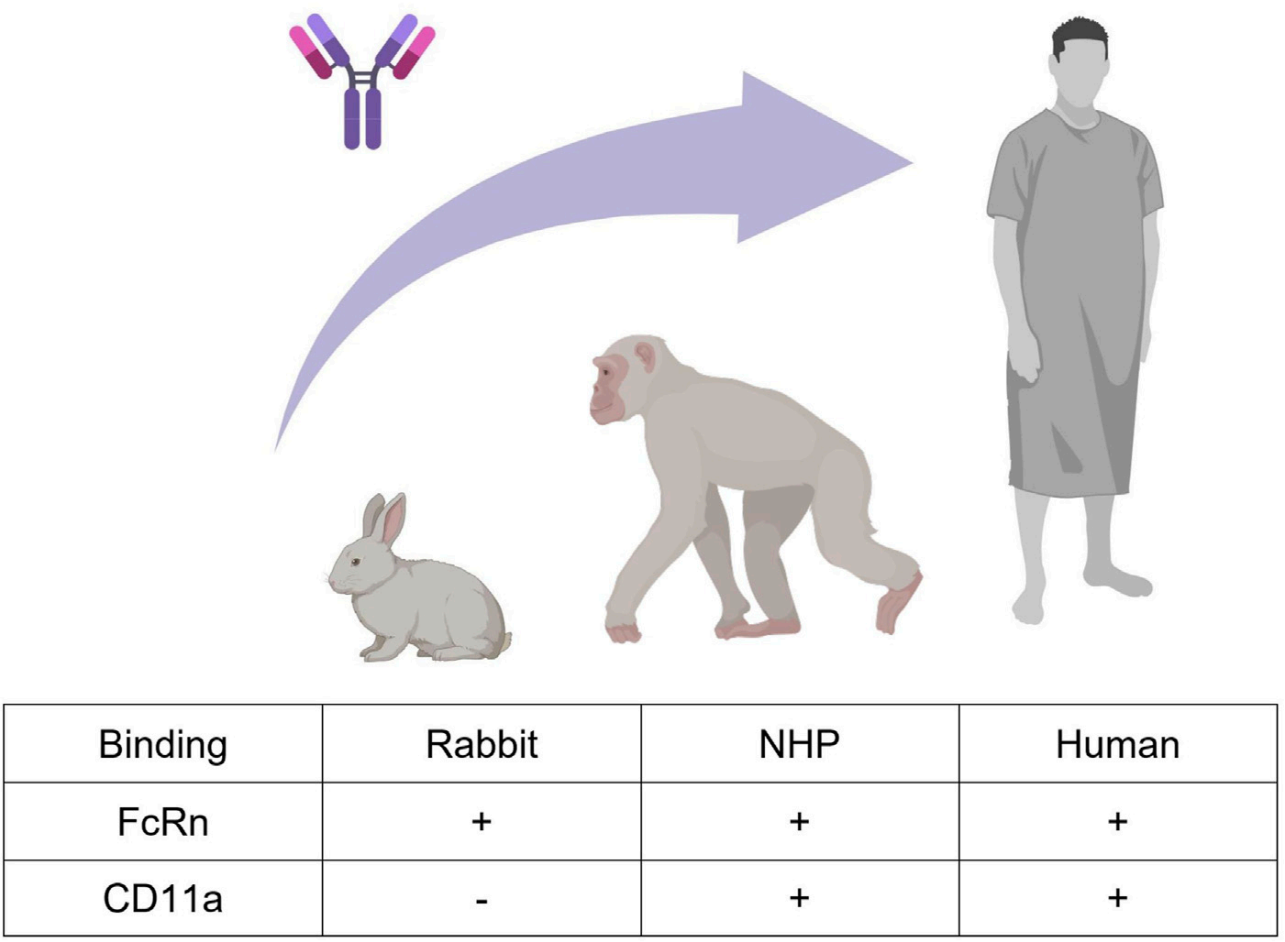
Efalizumab cross-species translation between rabbit, monkey, and human. Two mechanisms that highly influence the PK of large IgG molecules are FcRn-binding and target-binding. Efalizumab binds to rabbit, non-human primate (NHP) and human FcRn. Target-binding to CD11a is only present in NHP and human, but not in rabbit (Bauer et al., 1999; Mortensen et al., 2005; Mould and Green, 2010). Created with BioRender.com.

We here considered efalizumab (Mortensen et al., 2005), a humanized immunoglobulin G-1 (IgG1) monoclonal antibody formerly used in psoriasis therapy. It is directed against the CD11a subunit of leukocyte function-associated antigen 1 (LFA-1), an adhesion receptor expressed in leucocytes, and was developed as an immunosuppressant through inhibition of lymphocyte activation. The PK of efalizumab after intravenous administration has been studied in rabbits, NHPs and human psoriasis patients (Bauer et al., 1999; Gottlieb et al., 2000; 2002; Ng et al., 2005; Mould and Green, 2010; Chetty et al., 2015). In human, efalizumab PK was shown to exhibit a dose-dependent clearance. At 10 mg/kg efalizumab plasma half-life was determined to be 5.3 days, while at lower doses clearance was more rapid, resulting in a half-life of 0.4 days at 0.1 mg/kg (Bauer et al., 1999; Gottlieb et al., 2000). These dose-dependent pharmacokinetics are caused by target mediated drug disposition (TMDD). Concentration-time profiles of a drug, which undergoes TMDD, exhibit a characteristic shape. Four phases can be distinguished: A rapid initial drop where drug binds to the free target, a linear phase dominated by the endosomal degradation of the free drug, a transition phase where the target is no longer saturated and a terminal linear phase where the drug is mainly eliminated through the target (Peletier and Gabrielsson, 2012). In chimpanzees, non-linear pharmacokinetics have been observed as well, which is expected given that efalizumab binds to chimpanzees’ CD11a (Bauer et al., 1999). In rabbits on the other hand, efalizumab is not cross-reactive, and pharmacokinetics are therefore linear (Mortensen et al., 2005; Mould and Green, 2010). The non-saturable share of efalizumab clearance occurs via protein degradation in endosomes. Antibodies containing an Fc-part can be protected from endosomal clearance through binding to the neonatal fragment crystallizable receptor (FcRn). Unlike binding to CD11a, FcRn-binding is probably also relevant in rabbits, as it was observed *in-vitro* for different humanized IgG antibodies (Szikora et al., 2017). Figure 1 summarizes the described main processes governing efalizumab PK, which need to be considered and investigated for translation between the three analyzed species.

In the current study, we investigated the translatability of large-molecule PBPK models between rabbit, NHP and human. The therapeutic protein efalizumab was selected for this case-study, as time-resolved plasma PK data after intravenous administration were available in the literature for all three species. A full TMDD model was used to build models with physiologically interpretable parameters that can be evaluated against experimental values from literature (see Results). The assumption of similar target-binding and target internalization between species was explored, to gain insights into how these parameters might be translated for first in human predictions.

## 2 Methods

Rabbit, NHP and human plasma PK data after intravenous administration of efalizumab were obtained from literature (see Table 1). Mean data were digitized from the published figures with the WebPlotDigitizer tool (https://automeris.io/WebPlotDigitizer/). Data from Ng et al. (Ng et al., 2005) were not considered for model development as the concentration-time profile for the 0.6 mg/kg dose does not align well with the other observed data (see Supplementary Material). Also, data from Gottlieb et al. (Gottlieb et al., 2000; Gottlieb et al., 2002) were not used, as they only published data following multiple administration. PBPK models for efalizumab (rabbit, NHP and human) were built with the Open Systems Pharmacology Suite (OSP Suite, http://www.open-systems-pharmacology.org), Version 11.1. Its software tool PK-Sim allows the selection of different species, accounting for anatomical and physiological differences between them, such as organ volumes, blood flow rates and tissue composition. We recently evaluated the applicability of the rabbit model structure (Jairam et al., 2024). The standard individuals were created with the following body weights: human (male, 73 kg), monkey (52.8 kg), rabbit (3 kg). The model was fitted to the data using the Levenberg-Marquardt algorithm in the OSP suite. The PBPK models were validated by comparison of the predicted concentration-time profiles to the experimental observations. The OSP Suite-R package was used to generate concentration-time plots (OSP suite results were imported via the ospsuite package). Sensitivity analysis was performed for single parameters of the model (local sensitivity analysis). It provides information on how the variation of certain parameters contributes to the overall uncertainty of a model. The area under the curve (AUC) extrapolated to infinity was used as the PK parameter to compare sensitivities. Sensitivity was calculated as the ratio of the relative change of AUC and the relative variation of the input parameter according to the following equation:

$$S=\frac{∆AUC}{AUC}\cdot \frac{p}{∆p}$$

with AUC: simulated AUC with the original input parameter value, ΔAUC: change of simulated AUC, Δp: change of the input parameter value, p: input parameter value, and S: sensitivity.

**TABLE 1:** PK data of efalizumab used for model building.

Species	Doses	Source
Human	0.1; 0.3; 1.0; 3.0; 10 mg/kg	Bauer et al., 1999
Chimpanzee	8 mg/kg	Bauer et al., 1999
New Zealand White Rabbit	2 mg/kg	Mould and Green, 2010

Parameters related to target turnover, target-binding and FcRn-binding were selected for the analysis in order to quantify their relevance for cross-species translation. This included all estimated model parameters, as well as parameters which were assumed to have a strong influence on PK. Sensitivity was calculated in MoBi with a relative parameter perturbation of 1,000% (variation range = 10, maximum number of steps = 9).

## 3 Results

The goal of the current study was to investigate the translatability of large-molecule PBPK models between rabbit, NHP and human. We selected the therapeutic protein efalizumab for our case-study, as time-resolved data of plasma samples after intravenous administration were available in the literature for all three species. The developed models contained physiologically interpretable parameters that can be evaluated against experimental values and compared between species. In particular, parameters related to FcRn affinity, target-binding and target turnover and internalization were explored. As a first step, we developed and evaluated the rabbit model, before moving to a more complex model structure for NHP and human.

### 3.1 Model structure

PK-Sim’s PBPK model structure for large molecules was used as a basis for model development (Niederalt et al., 2018). It includes the typical mechanisms of large molecule disposition which is governed by the following physiological and biochemical processes:

- Transcapillary exchange of the drug between plasma and interstitial spaces, described by the two-pore formalism (Rippe and Haraldsson, 1987; Rippe and Haraldsson, 1994). The drug passes through small and large vascular pores by convection and diffusion. This is influenced by the hydrodynamic radius of the drug, the radii of the vascular pores, the fraction of large pores, and the hydraulic conductivity of the vascular endothelium.
- Transport into lymph vessels by convection (modeled via reflection coefficients).
- Uptake of the drug into endothelial cells via pinocytosis.
- Degradation of the free drug in endosomes of endothelial cells (fixed degradation rate constant in PK-Sim).
- Protection from endosomal degradation by binding of drug to FcRn, characterized by the dissociation rate constant k_off, FcRn_, the association rate constant k_on,FcRn_ and the equilibrium dissociation constant K_D,FcRn_ = k_off, FcRn_/k_on,FcRn_).

The OSP modeling software tool MoBi was used for an extension of the NHP and human PBPK including the TMDD processes given below:

- Target synthesis and internalization, modeled via the steady state target concentration (T_SS_) and a target internalization rate constant (k_int,T_).
- Reversible binding of drug to free target (characterized by the rate constants k_on_ and k_off_), forming a drug-target-complex.
- Internalization of the drug-target-complex, modeled by the rate constant k_int,C_.

The following equations were used to account for these processes:

$$\frac{dD}{dt}=-k_{on}\cdot D\cdot T+k_{off}\cdot C$$

$$\frac{dT}{dt}=k_{\mathrm{int},T}\cdot \left(T_{SS}-T\right)\;–\;k_{on}\cdot D\cdot T+k_{off}\cdot C$$

$$\frac{dC}{dt}=k_{on}\cdot D\cdot T\;–\;k_{off}\cdot C\;–\;k_{\mathrm{int},C}\cdot C$$

with C: drug-target-complex, D: drug, k_off_: dissociation rate constant, k_on_: association rate constant (k_on_ = k_off_/K_D_, using the equilibrium dissociation constant K_D_), k_int,C_: internalization rate constant of the drug-target-complex, k_int,T_: internalization rate constant of the target, T: target, and T_SS_: steady state target concentration.

**FIGURE 2:**
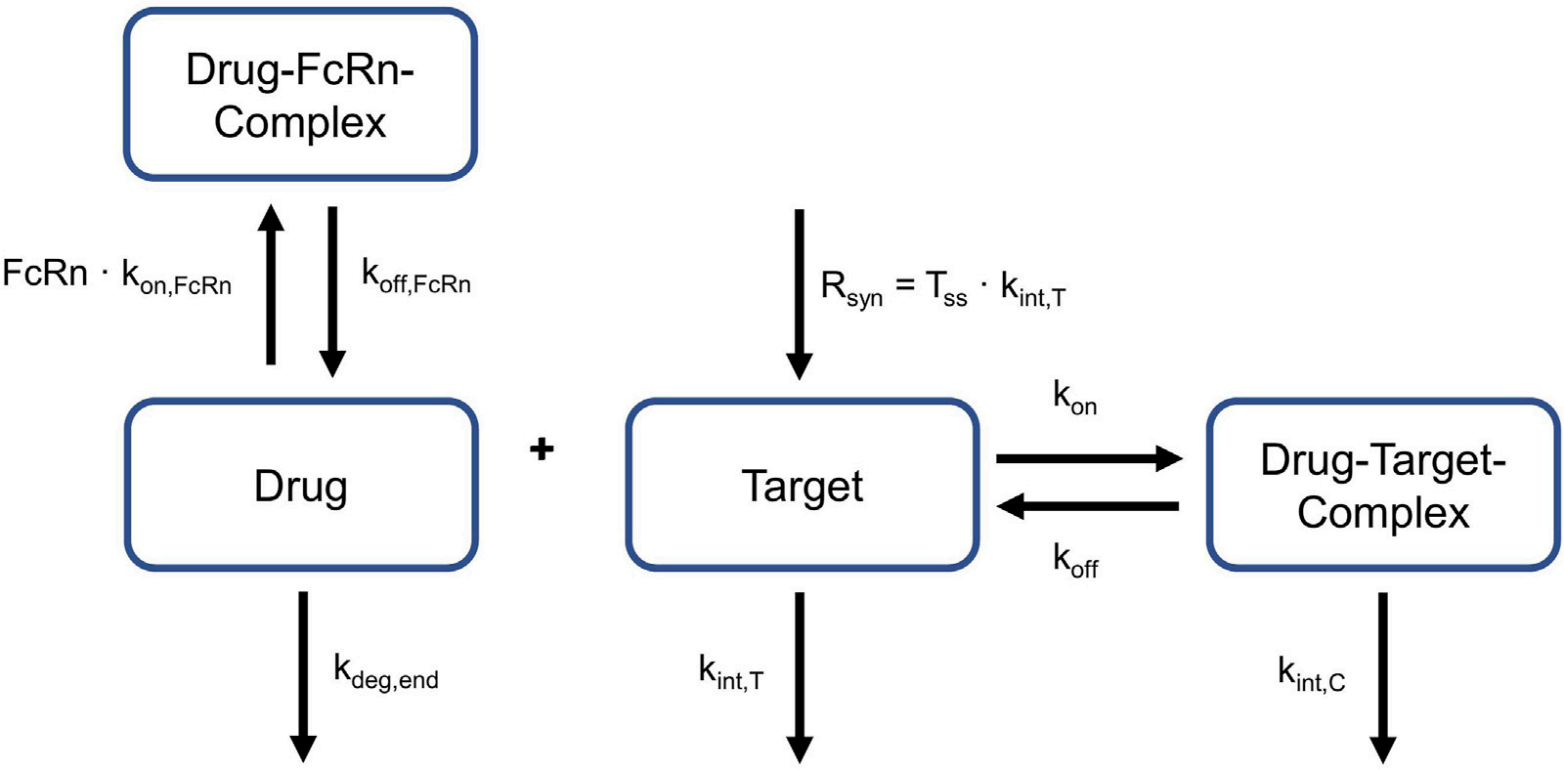
Relevant reactions for target- and FcRn-binding. Drug can bind to and dissociate from target and FcRn. Target is produced constantly. Target and drug-target-complex are internalized with different rates. Target turnover and target binding is only applicable to NHP and humans, not to rabbits. FcRn: neonatal Fc Receptor, k_deg,end_: degradation rate constant in endosomes, k_int,C_:internalization rate of the drug-target-complex, k_int,T_: internalization rate of the target, k_off_ dissociation rate constant of the drug-target-complex, k_off, FcRn_: dissociation rate constant of the drug-FcRn-complex, k_on_: association rate constant of the drug-target-complex, k_on,FcRn_: association rate constant of the drug-FcRn-complex, R_syn_: zero-order synthesis rate of the target, T_ss_: target concentration in steady state.

These equations given above represent a full TMDD model, i.e., they contain physiologically interpretable rate constants, rather than lumping them together in one parameter (Peletier and Gabrielsson, 2012). This allows for a comparison of single parameters between species. More precisely, it was possible to explore if values for k_int,C_, k_int,T_, k_off_ and T_SS_ can be translated between NHP and human. The relevant processes for target-binding and FcRn-binding are schematically displayed in Figure 2. This is a simplified representation of what was embedded into the PBPK model structures of NHP and human, the complete implementation is provided in Niederalt et al. (Niederalt et al., 2018).

### 3.2 Model parameters

The hydrodynamic radius of efalizumab was assumed to be identical between species, as it is a physico-chemical parameter that depends on the drug only. Thus, the same value of 3.51 nm was used for all species. It was calculated with the formula (Hutton-Smith et al., 2016):

$$R_{hyd}={\left(\frac{3vMW}{4\pi N_{A}}\right)}^{1/3}$$

With MW: molecular weight, N_A_: Avogadro’s number, and *v*: partial specific volume of protein, taken as 0.73 cm^3^/g. K_D,FcRn_ of the human model was fixed to the value provided by the OSP suite (Niederalt et al., 2018). It was shown previously that FcRn-binding kinetics differ between species (Abdiche et al., 2015). Thus, K_D,FcRn_ for the rabbit and NHP model were not taken from the human model, but estimated. The association rate constant k_on,FcRn_ as well as the degradation rate constant in endosomes k_deg,end_ were taken as provided in the OSP suite. Furthermore, the CD11a expression reference concentration in the organ with maximal target expression (only relevant for NHP and human) was assumed to be the same in NHP and human. The OSP suite includes publicly available data of relative gene expression across different organs and tissues which can be added to a PBPK model (Meyer et al., 2012). The relative expression of CD11a was taken from the human gene expression database for both, the NHP and human model. The Expressed Sequence Tags (EST) gene database was selected and CD11a was assumed to be expressed in plasma and interstitial space, according to the location of leucocytes. Efalizumab binding to CD11a was studied *in-vitro* for NHP and human. Bauer et al. reported comparable K_D_ values for NHP and human of 0.99 nM and 0.77 nM, respectively (Bauer et al., 1999). K_D_ values were fixed to these literature values in the NHP and human models. As no literature was found on the dissociation rate constants, the same value for k_off_ was assumed for both models. Target-binding of mAbs is generally thought to be similar between NHPs and humans due to the high sequence homology (Germovsek et al., 2021). Furthermore, the internalization processes of CD11a and drug-bound CD11a, and with that, the values for k_int,T_ and k_int,C_, respectively, were assumed to be similar in NHPs and humans. CD11a reference concentration, k_off_, k_int,T_ and k_int,C_ were estimated. During the estimation procedure, k_int,C_ was restricted to differ from k_int,T_ by a scaling factor between 0.1 and 10. This allowed the drug-induced change in the internalization rate to remain in a similar range as observed by Bauer et al. and Ng et al., who estimated a maximum of 3- (Bauer et al., 1999) or 5-times (Ng et al., 2005) increased internalization rate induced by efalizumab-binding in humans. For the human model, infusion time was estimated with boundaries between 60 and 180 min, as the exact values were not provided in the literature source.

### 3.3 Modeling outcome

**FIGURE 3:**
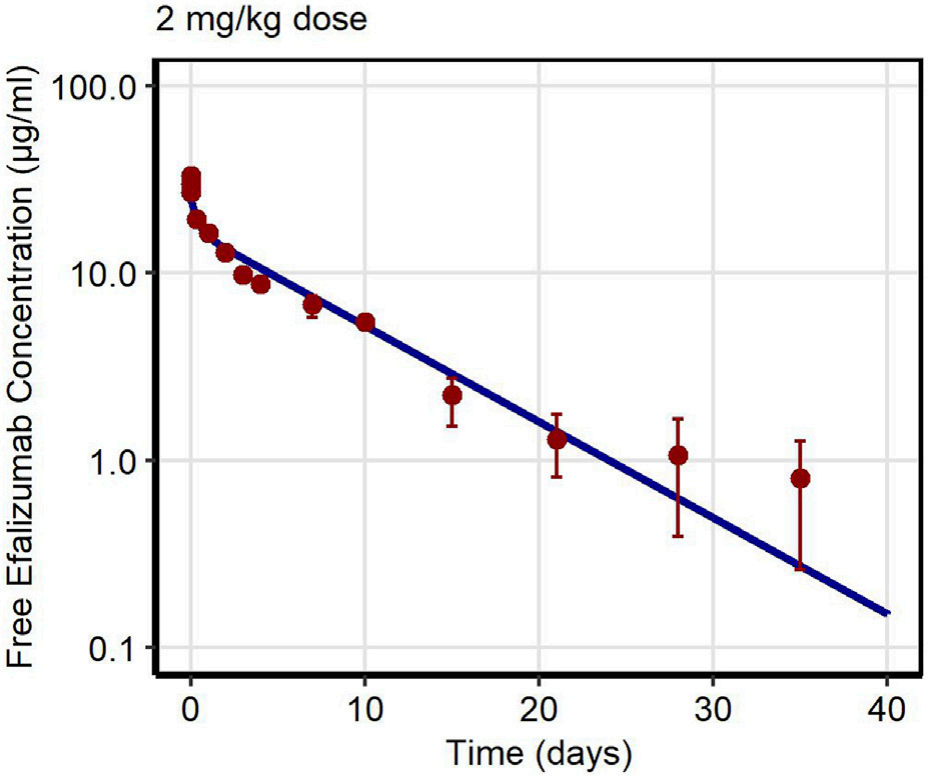
Free efalizumab concentrations over time in rabbit. Line: Simulation, points: observed data (Mould and Green, 2010).

**FIGURE 4:**
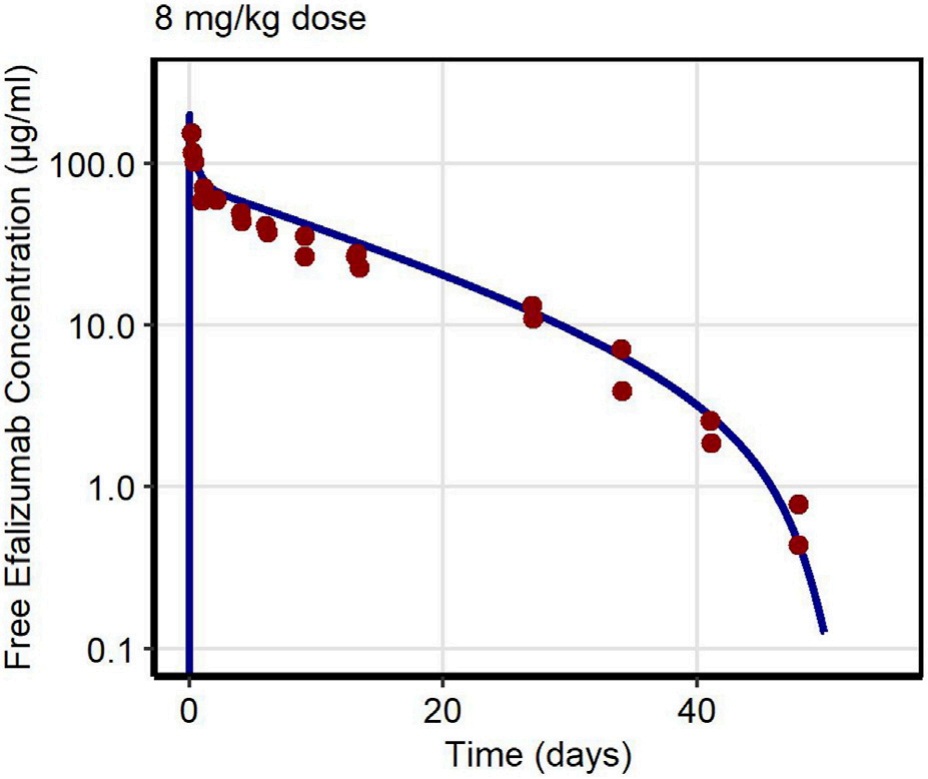
Free efalizumab concentrations over time in NHP. Line: Simulation, points: observed data (Bauer et al., 1999).

**FIGURE 5:**
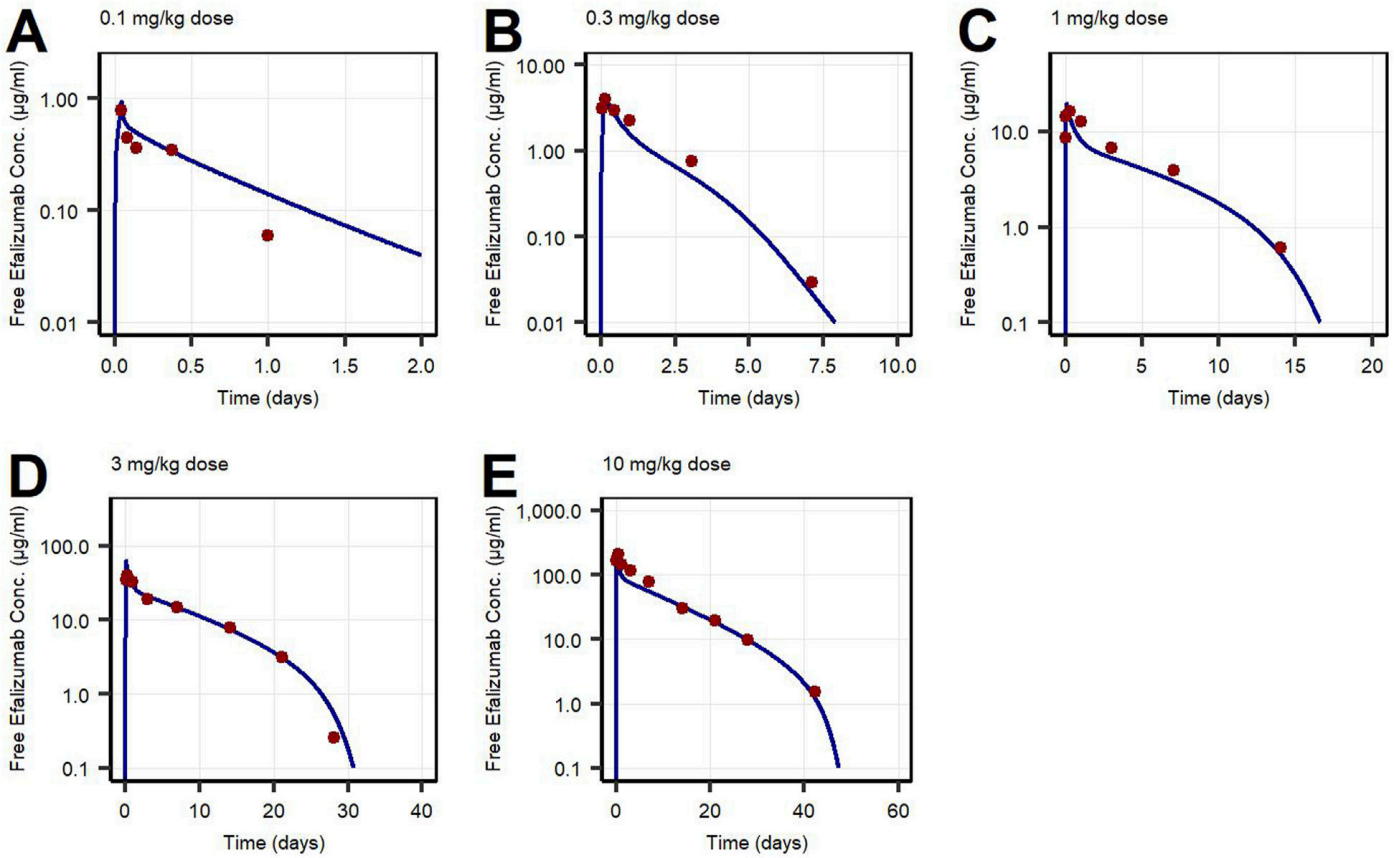
Free efalizumab concentrations over time in human following intravenous administration of **(A)** 0.1 mg/kg, **(B)** 0.3 mg/kg, **(C)** 1.0 mg/kg, **(D)** 3.0 mg/kg, **(E)** 10 mg/kg. Lines: Simulations, points: observed data (Bauer et al., 1999).

**FIGURE 6:**
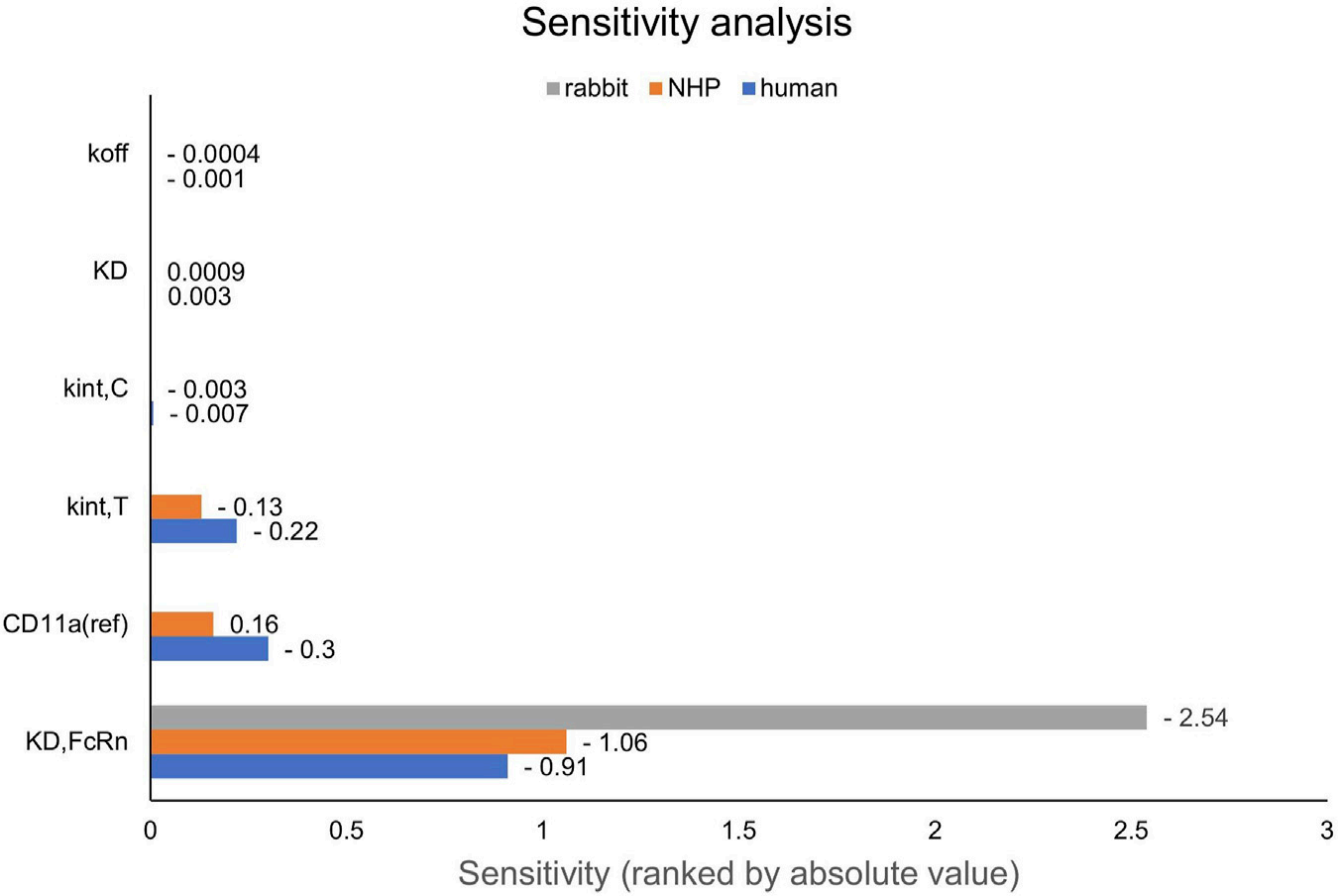
Local sensitivity analysis of the rabbit, NHP and human models. Sensitivity of the model to single parameters, ranked by their impact on the predicted value of AUC extrapolated to infinity. CD11a (ref): CD11a reference concentration, K_D_: equilibrium dissociation rate constant of the drug-target-complex, K_D,FcRn_: equilibrium dissociation rate constant of the drug-FcRn-complex, k_int,C_: internalization rate of the drug-target-complex, k_int,T_: internalization rate of the target, k_off_: dissociation rate constant of the drug-target-complex, NHP: non-human primate.

First, PK data of efalizumab in rabbits were considered (Mould and Green, 2010). Concentration–time profiles following intravenous administration of 2 mg/kg efalizumab in rabbits could be adequately described by the model without target-binding (Figure 3), even though only one parameter (KD_FcRn_) was allowed to vary during the estimation process. After demonstrating the applicability of the structure of the rabbit model, we moved on to the NHP and human models, where different FcRn binding kinetics and additional TMDD processes were expected. The models with target-binding were able to describe the concentration–time profiles of efalizumab in NHP for 8 mg/kg intravenous administration (Figure 4) and in human for 0.1, 0.3, 1.0, 3.0 and 10 mg/kg intravenous efalizumab administration (Figure 5). Notably, the model structure could well capture the PK behavior for doses over two orders of magnitude. Table 2 summarizes the estimated and fixed parameters of the three developed PBPK models. All estimated parameters were carefully selected, and the parameter bounds were set to be narrow. The same values of CD11a reference concentration, koff, kint,T and kint,C were assumed for NHP and human and the deviation between kint,C and kint,T was limited. Estimated infusion times for the human administrations were 180 min for all doses, except for the lowest dose of 0.1 mg/kg (60 min). Figure 6 shows the results of the local sensitivity analyses of the three models (for the human model the 3 mg/kg dose is shown, results for the other doses can be found in the Supplementary Material). All models show highest sensitivity for K_D,FcRn_. This suggests that endosomal clearance is the most relevant process to determine efalizumab PK after administration of the given doses. For the human and NHP models, CD11a reference concentration and k_int,T_ are moderately sensitive parameters, while target-binding parameter values and k_int,C_ have no significant impact on the predicted AUC extrapolated to infinity.

**TABLE 2:** Parameters of efalizumab PBPK models.

Parameter	Rabbit	NHP	Human	Source	Description
K_D,FcRn_ [μM]	6.51	0.49	0.63	Human: Niederalt et al., 2018 NHP and rabbit: estimated	Equilibrium dissociation constant of efalizumab binding to FcRn
CD11a reference concentration [μM]	—	0.12^a^	0.12^a^	Estimated	Concentration in organ with maximal expression (=gonads)
k_int,T_ [h^−1^]	—	0.005^a^	0.005^a^	Estimated	Internalization rate constant of CD11a
k_int,C_ [h^−1^]	—	0.05^a^	0.05^a^	Estimated	Internalization rate constant of the efalizumab-CD11a-complex
K_D_ [nM]	—	0.99^b^	0.77^b^	Bauer et al., 1999	Equilibrium dissociation constant of efalizumab binding to CD11a
k_off_ [h^−1^]	—	0.33^a^	0.33^a^	Estimated	Dissociation rate constant of the efalizumab-CD11a-complex
MW [kDa]	149	149	149	Boehncke, 2007	Molecular weight
R_hyd_ [nm]	3.51	3.51	3.51	Calculated	Hydrodynamic radius
k_ass_ [µM^−1^ · min^−1^]	0.87	0.87	0.87	PK-Sim default	Association rate constant of efalizumab binding to FcRn

^a^ Same value assumed for NHP, and human.

^b^ *In-vitro* measurement.

## 4 Discussion

Mechanistic PBPK models of efalizumab in rabbit, NHP and human were built and successfully evaluated. Altogether, the PBPK models of efalizumab in the three species adequately describe the available experimental data. Moreover, the models allow for a mechanistic comparison of the physiological processes underlying large molecule PK in rabbit, NHP and human. The use of a PBPK platform makes the work easily reproducible and enables the applicability of the models for further investigation of cross-species differences. Furthermore, the newly developed models were applied to analyze differences in single parameters, as such reflecting specific physiological processes, and to explore the assumption of similar target-binding and target internalization between species. Thus, this work provides valuable insights into the inter-species translatability of large-molecule PBPK models.

Concentration–time profiles following intravenous administration were adequately described by the three models, with only few deviations from the observed data. For the rabbit model, the predictions were within the error bars, however, two data points after 28 days post-dose were under-predicted. This might be due to a limited accuracy of the experiment as the drug concentrations approach the lower limit of quantification for the assay (0.025 μg/mL). An alternative interpretation could be binding of efalizumab in peripheral tissues and subsequent redistribution. However, no Supplementary Material in literature could be found. Therefore, in order to maintain simplicity, the model structure was kept as basic as possible. Additionally, the original data source (Mould and Green, 2010) states that the PK profile is linear. For the human model, the observed concentration at 1 day after the 0.1 mg/kg dose was over-predicted. This can be attributed to the internalization rate which highly influences the last phase of a classical TMDD profile. k_int,T_ and k_int,C_ were restricted to a certain range (maximal 10-fold deviation) during parameter identification. Although relaxing this restriction would result in a better fit for the 0.1 mg/kg dose profile, it would be less consistent with the models proposed in literature. Bauer et al. (Bauer et al., 1999) estimated a maximal 3-fold increase of internalization rate induced by efalizumab for human and a maximal 4.5-fold increase for monkey. According to the model of Ng et al. (Ng et al., 2005), internalization is maximal 5-times increased upon efalizumab binding in humans.

The K_D,FcRn_ for human was fixed to the value of 0.63 µM provided by OSP suite (Niederalt et al., 2018). The measured *in-vitro* FcRn affinity for efalizumab is lower, with a K_D,FcRn_ of 1.8 µM (Chung et al., 2019). Also, Suzuki et al. (Suzuki et al., 2010) predicted a lower affinity with a linear regression model. The estimated K_D,FcRn_ for NHP was 0.49 µM, which corresponds to a 22% higher affinity compared to binding to the human FcRn. It was shown in *in-vitro* experiments that cynomolgus monkey FcRn binds to humanized IgG1 with a higher affinity than human FcRn. A 50%–60% higher affinity was reported from surface plasmon resonance biosensor assays (Dall’Acqua et al., 2006; Abdiche et al., 2015). Other studies also observed higher affinity in NHP compared to human using a biolayer interferometry assay. They report a 60%–75% higher affinity of humanized IgG1 for cynomolgus monkey compared to human FcRn (Deng et al., 2010; Neuber et al., 2014). As intravenous data from chimpanzees and not cynomolgus monkeys were used for this model, the differences in affinity may not be fully translatable, however there might be a similar trend towards higher FcRn affinity for humanized IgG1. The estimated K_D,FcRn_ for the rabbit model was 6.51 µM, which is about 10-fold larger than for human. *In-vitro* experiments have shown that rabbit FcRn has a lower affinity for humanized IgG1. However, the study reports only a 2-fold higher K_D,FcRn_ for rabbit compared to human (Szikora et al., 2017). Even though these results are in line with the trends in literature, the interpretation has to be treated with caution. The estimation of K_D,FcRn_ depends on a number of parameters, like the endosomal FcRn concentration and k_on,FcRn_, as well as the endogenous IgG concentration and related binding kinetics. For these parameters the default values of PK-Sim were used and potential errors in these default parameter values are passed on to the K_D,FcRn_ estimate. Especially for the rabbit model, the default values are not validated sufficiently: for human and monkey, PK-Sim incorporates species specific values for FcRn concentration, endogenous IgG concentration and the affinity of endogenous IgG to FcRn, while the rabbit model uses only “reasonable values” (Open Systems, 2018). The NHP and human models suggest an internalization rate of CD11a of 0.005 h^-1^. This value is lower than predicted by the models of Bauer et al. and Ng et al. which were around 0.02 h^-1^ (Bauer et al., 1999; Ng et al., 2005). The internalization rate of the efalizumab-CD11a-complex was estimated to be 0.05 h^-1^. Coffey et al. (Coffey et al., 2004) measured the internalization of antibody-linked efalizumab into human blood T cells in the presence of concanamycin A (to inhibit cellular clearance). They observed that 48% of the drug was internalized by the cells after 24 h. Assuming a linear uptake rate, this corresponds to an internalization rate of 0.02 h^-1^. Thus, the estimated value of k_int,C_ has the same dimension as the *in-vitro* measurement. Bauer et al. and Ng et al. estimate a similar value for the internalization rate of the target, not the drug-target-complex (Bauer et al., 1999; Ng et al., 2005). Here, the estimated CD11a internalization is 10-fold increased upon efalizumab binding, which is higher than their estimations. Bauer et al. estimated a maximum 3-fold increase of internalization rate induced by efalizumab for human and a maximum 4.5-fold increase for NHP (Bauer et al., 1999). According to the model of Ng et al., internalization is 5-times increased at the most upon efalizumab binding in humans (Ng et al., 2005).

It is worth noticing however, that the monkey model structure utilized in PK-Sim was initially designed for a lightweight macaque monkey (Willmann et al., 2007). As in PBPK the body weight consists of the sum of all organ weights, it is difficult to apply allometric scaling. When adjusting the body weight to match that of a chimpanzee, a relatively straightforward method is employed by the software: all organs are uniformly scaled using the same factor, rather than an allometric exponent. This linear approach could be improved considering the significant body weight difference. It may be advantageous to extend the OSP suite by a chimpanzee individual, similar to what was done for dogs and beagles (Open Systems, 2018). Overall, the data of all three species were successfully described, given the assumptions of:

- similar CD11a expression in NHP and human.
- similar internalization kinetics of free and drug-bound CD11a in NHP and human.
- comparable binding kinetics of efalizumab to CD11a in NHP and human, no binding in rabbit.
- different binding affinities of efalizumab to FcRn in rabbit, NHP and human.

These results suggest that, when translating the efalizumab model from rabbit to primates, FcRn affinity has to be increased and TMDD processes need to be added. This finding may hold true for other IgG types as they also show lower *in-vitro* affinity to rabbit FcRn compared to human FcRn (Szikora et al., 2017). However, for other Fc containing therapeutics this might not be the case. As the rabbit model for efalizumab cannot give any insight into TMDD related parameters it may not be very useful for a translation to human. Thus, we conclude that rabbit is less relevant as an animal model for compounds that are not cross-reactive. While this study focused on rabbits and NHPs as animal models for translation, rodents are also often used for PK assessments. It has been demonstrated that mouse and rat PK models can be translated to humans successfully (Wang et al., 2016). However, as in the case of efalizumab in rabbits, drugs are often not cross-reactive in rodents (Germovsek et al., 2021). Consequently, in such instances, determining target binding parameters becomes challenging.

NHPs on the other hand, are more relevant when extrapolating to human. The presented results suggest, that all TMDD related parameters (target binding, internalization, expression) can be translated from NHP to human and only the FcRn affinity should be decreased. However, this must be treated with caution as the available data from NHPs for efalizumab are limited to only one dose. In the human study, the wide range of administered doses gives a good picture of the different phases of TMDD (rapid drop when reaching low concentrations, rapid drop from the beginning at the lowest dose). The sensitivity of the NHP and human models to the values of k_int,C_ and target-binding parameters is low for the doses shown in Figure 6, but increases for lower doses (see Supplementary Material). A study design with different doses in NHP would be valuable to confirm the model parameter values identified in this study.

Target-binding constants are generally assumed to be comparable in NHPs and humans (Deng et al., 2012; Germovsek et al., 2021). Even though the *in-vitro*/*in-vivo* relationship of binding parameters can differ substantially between species (Gabrielsson and Hjorth, 2023), the low sensitivity of the models to values of K_D_ and k_off_ suggests that a small deviation between species has no big impact on the model predictions, thus the assumption of similar binding rate constants between NHPs and humans seems to be of little consequence, even if it might not be valid.

Unlike target-binding constants, target expression and turnover are more likely to differ between species (Deng et al., 2012; Gabrielsson and Hjorth, 2023). Our results suggest that CD11a reference concentration, k_int,T_ and k_int,C_ can be translated between NHP and human. However, parameters may have been not unambiguously identifiable for the NHP model because only one dose group was available. As shown in Figure 6, the monkey model is less sensitive to the respective parameters than the human model. When estimating TMDD related parameters (CD11a reference concentration, k_off_, k_int,T_ and k_int,C_) using only the NHP data, the human data were not well described after transferring the NHP-derived parameters to the human model (results not shown). Thus, it could not be demonstrated that human PK is predictable by a translation from NHP, however, the fact that a common parameter set achieved from a simultaneous fit of NHP and human data (6 dose groups in total) can describe both NHP and human data, suggests that this may be possible with a larger NHP dataset used for parameter identification.

As an alternative to prediction of human pharmacokinetics from preclinical studies, one could question the need for animal models in general, if there is not enough data collected to estimate relevant parameters in these preclinical studies and if the most sensitive parameters cannot be translated between species. Sufficient human *in-vitro* data on key parameters such as target expression, binding kinetics and internalization rates might give more insightful information than limited animal data. While binding kinetics are already commonly tested *in-vitro*, assays for internalization rates and target expression are still rare although they are of high value considering their impact on model predictions. With information on target expression and internalization rates, it may be possible to develop comprehensive human PBPK models without the need for animal testing.

Apart from the discussed limitations, the developed models are, to the best of our knowledge, the first PBPK models used to analyze efalizumab PK across three species. The use of a full (instead of a reduced) TMDD structure enables the comparison of physiologically interpretable parameters between NHP and human. The assumption of similar target-binding and target (-complex) internalization in NHP and human was confirmed for the available data. The presented models can be used to further investigate cross-species differences if additional data, preferably in NHPs, become available, or when other species of interest emerge. Finally, a growing understanding of mAb disposition across species can improve first in human predictions and enable more adequate dosing and sampling designs in early clinical trials.

## Data Availability

Publicly available datasets were analyzed in this study. This data can be found here: Bauer et al., 1999. https://pubmed.ncbi.nlm.nih.gov/10826130/ Mould and Green, 2010 https://pubmed.ncbi.nlm.nih.gov/20055530/.
