# Supplementary figures and images for: PBPK-based translation from preclinical species to humans for the full-size IgG therapeutic efalizumab
Source: Front Pharmacol. 2024 Oct 1;15:1418870. doi: 10.3389/fphar.2024.1418870 (PMC11473394; doi:10.3389/fphar.2024.1418870)

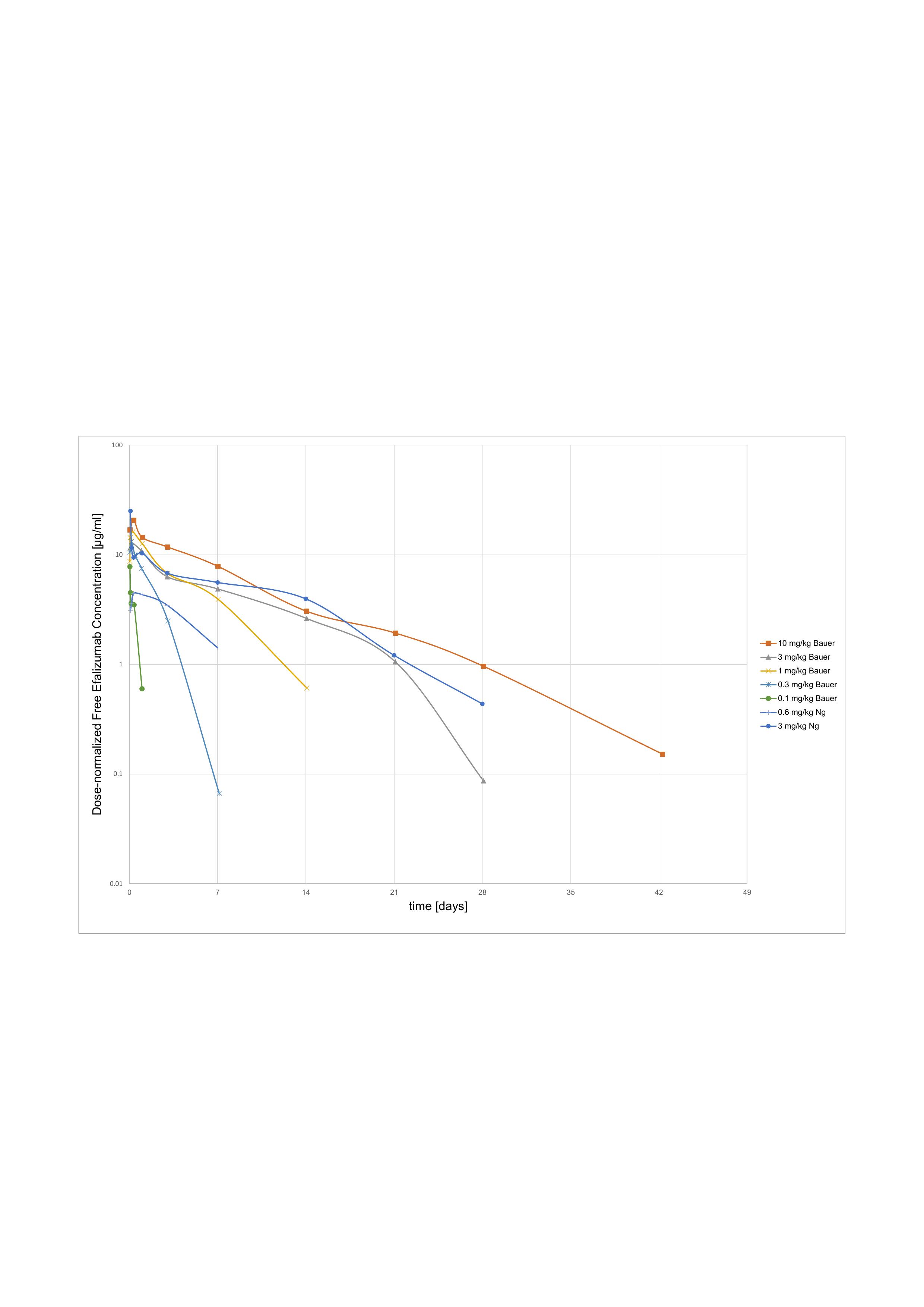

Supplement: Supplementary file 1 [file Image1.JPEG]
