# Supplementary material for: PBPK-based translation from preclinical species to humans for the full-size IgG therapeutic efalizumab
Source: Front Pharmacol. 2024 Oct 1;15:1418870. doi: 10.3389/fphar.2024.1418870 (PMC11473394; doi:10.3389/fphar.2024.1418870)
Supplement: Supplementary file 2 [file DataSheet1.pdf]

Supplementary Material

PBPK-based translation from preclinical species to humans for the full-size IgG therapeutic efalizumab

Maria Franz[1*], Ravi Kumar Jairam[2], Lars Kuepfer[2], Nina Hanke[1]

[1]Translational Medicine and Clinical Pharmacology, Boehringer Ingelheim Pharma GmbH & Co. KG, 88400 Biberach an der Riß, Germany

[2]Institute for Systems Medicine with Focus on Organ Interaction, University Hospital RWTH Aachen, 52062 Aachen, Germany

*** Correspondence:**
Maria Franz
maria.franz@boehringer-ingelheim.com

1 Supplementary Figures and Tables

1.1 Supplementary Figures

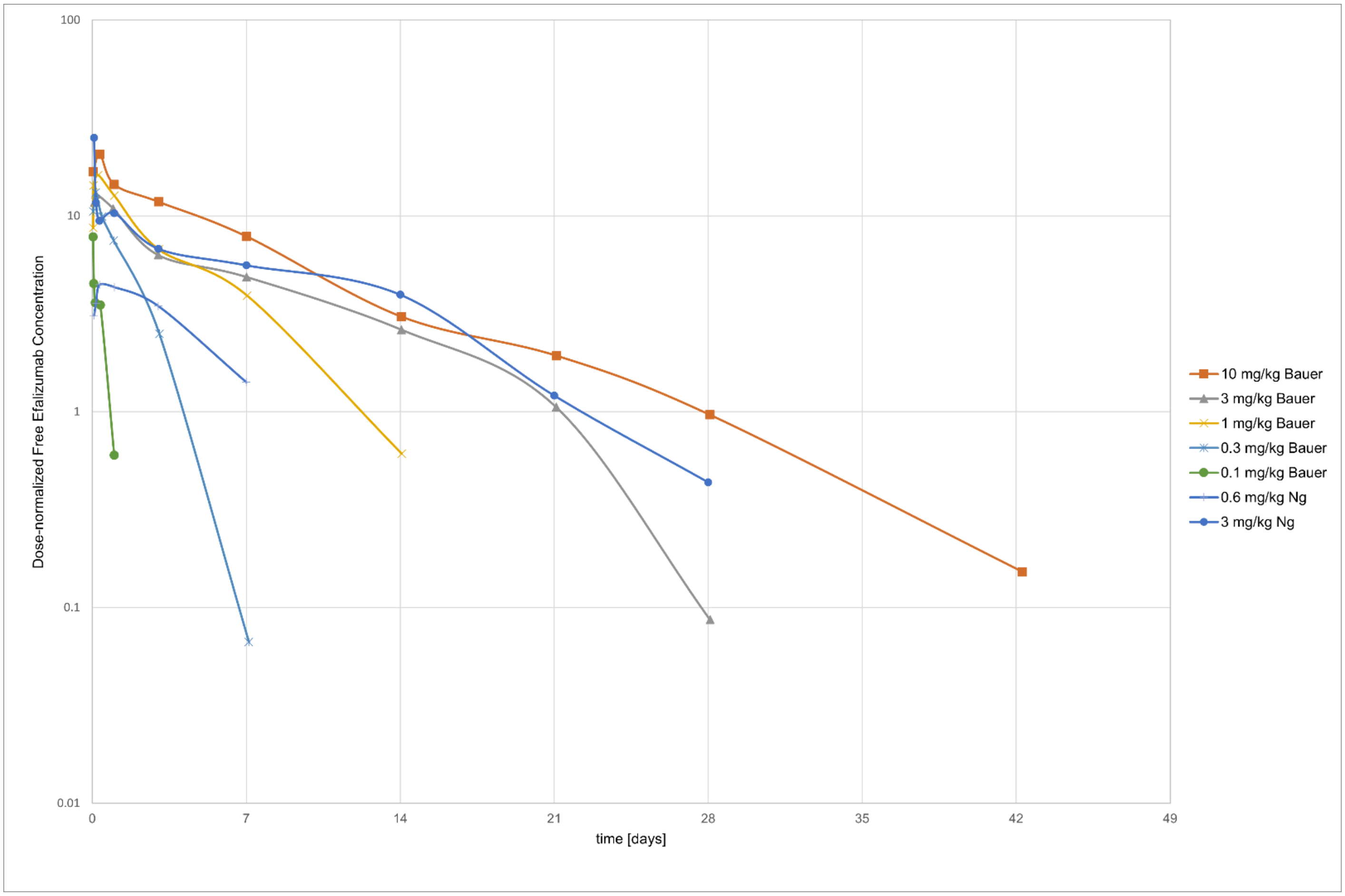

Supplementary Figure 1. Dose-normalized plot of the observed plasma efalizumab concentrations following intravenous administration in human. Data were obtained from literature (Bauer et al., 1999; Ng et al., 2005). Data from Ng et al. were not considered for model development as the concentration-time profile for the 0.6 mg/kg dose does not align well with the other observed data (early measurements are not lying between the observations of 0.3 mg/kg dose and 1 mg/kg dose).

1.2 Supplementary Tables

Supplementary Table 1. Local sensitivity analysis of the human efalizumab PBPK model performed in OSP suite. CD11a(ref) CD11a reference concentration, K_D equilibrium dissociation rate constant of the drug-target-complex, $K_{D,FcRn}$ equilibrium dissociation rate constant of the drug-FcRn-

complex, $k_{int,C}$ internalization rate of the drug-target-complex, $k_{int,T}$ internalization rate of the target, k_{off} dissociation rate constant of the drug-target-complex.

Parameter	AUC extrapolated to infinity, dose 0.1 mg/kg	AUC extrapolated to infinity, dose 0.3 mg/kg	AUC extrapolated to infinity, dose 1 mg/kg	AUC extrapolated to infinity, dose 3 mg/kg	AUC extrapolated to infinity, dose 10 mg/kg
$K_{D,FcRn}$	- 0.04	- 0.15	- 0.5	- 0.91	- 1.25
CD11a(ref)	- 5.55	- 2.15	- 1.23	- 0.3	- 0.12
$k_{int,T}$	- 0.13	- 0.34	- 0.5	- 0.22	- 0.09
$k_{int,C}$	- 0.82	- 0.2	- 0.0095	- 0.0075	- 0.0019
k_{off}	- 0.11	- 0.03	- 0.011	- 0.0011	- 0.00026
K_D	0.46	0.07	0.024	0.0026	0.00062

2 References

Bauer, R. J., Dedrick, R. L., White, M. L., Murray, M. J., and Garovoy, M. R. (1999). Population Pharmacokinetics and Pharmacodynamics of the Anti-CD11a Antibody hu1124 in Human Subjects with Psoriasis. *J Pharmacokinet Biop* 27, 397–420. doi: 10.1023/a:1020917122093.

Ng, C. M., Joshi, A., Dedrick, R. L., Garovoy, M. R., and Bauer, R. J. (2005). Pharmacokinetic–Pharmacodynamic–Efficacy Analysis of Efalizumab in Patients with Moderate to Severe Psoriasis. *Pharmaceut Res* 22, 1088–1100. doi: 10.1007/s11095-005-5642-4.
